# Providing insight into the incubation period of *Mycobacterium ulcerans* disease: two case reports

**DOI:** 10.1186/s13256-019-2144-2

**Published:** 2019-07-18

**Authors:** Y. A. Amoako, M. Frimpong, D. O. Awuah, G. Plange-Rhule, E. Boakye-Yiadom, B. Agbavor, F. Sarpong, H. Ahor, E. Adu, K. G. Danso, M. K. Abass, K. Asiedu, M. Wansbrough-Jones, R. O. Phillips

**Affiliations:** 1 Komfo Anokye Teaching Hospital, Kumasi, Ghana; 2 Kumasi Centre for Collaborative Research in Tropical Medicine, Kwame Nkrumah University of Science and Technology, Kumasi, Ghana; 3 School of Medicine and Dentistry, Kwame Nkrumah University of Science and Technology, Kumasi, Ghana; 4 Agogo Presbyterian Hospital, Agogo, Ghana; 5 Global Buruli Ulcer Initiative, WHO, Geneva, Switzerland; 6 Institute of Infection and Immunity, St George’s University of London, London, UK

**Keywords:** Buruli ulcer, Neonate, Incubation period, Ghana

## Abstract

**Background:**

Buruli ulcer caused by *Mycobacterium ulcerans* is endemic in parts of West Africa and is most prevalent among the 5–15 years age group; Buruli ulcer is uncommon among neonates. The mode of transmission and incubation period of Buruli ulcer are unknown. We report two cases of confirmed Buruli ulcer in human immunodeficiency virus-unexposed, vaginally delivered term neonates in Ghana.

**Case presentation:**

Patient 1: Two weeks after hospital delivery, a baby born to natives of the Ashanti ethnic group of Ghana was noticed by her mother to have a papule with associated edema on the right anterior chest wall and neck that later ulcerated. There was no restriction of neck movements. The diagnosis of Buruli ulcer was confirmed on the basis of a swab sample that had a positive polymerase chain reaction result for the IS2404 repeat sequence of *M. ulcerans*. Patient 2: This patient, from the Ashanti ethnic group in Ghana, had the mother noticing a swelling in the baby’s left gluteal region 4 days after birth. The lesion progressively increased in size to involve almost the entire left gluteal region. Around the same time, the mother noticed a second, smaller lesion on the forehead and left side of neck. The diagnosis of Buruli ulcer was confirmed by polymerase chain reaction when the child was aged 4 weeks. Both patients 1 and 2 were treated with oral rifampicin and clarithromycin at recommended doses for 8 weeks in addition to appropriate daily wound dressing, leading to complete healing. Our report details two cases of polymerase chain reaction-confirmed Buruli ulcer in children whose lesions appeared at ages 14 and 4 days, respectively. The mode of transmission of *M. ulcerans* infection is unknown, so the incubation period is difficult to estimate and is probably dependent on the infective dose and the age of exposure. In our study, lesions appeared 4 days after birth in patient 2. Unless the infection was acquired *in utero*, this would be the shortest incubation period ever recorded.

**Conclusions:**

Buruli ulcer should be included in the differential diagnosis of neonates who present with characteristic lesions. The incubation period of Buruli ulcer in neonates is probably shorter than is reported for adults.

## Background

Buruli ulcer (BU) caused by *Mycobacterium ulcerans* is endemic in parts of West Africa [1, 2]. The causative organism produces the toxin called *mycolactone* [3], which has been shown to be cytotoxic to a range of immune and nonimmune cells [4]. Although the toxin mediates a predominantly necrotizing cutaneous and subcutaneous tissue infection, it may occasionally involve deeper structures such as the bone, causing osteomyelitis. BU begins as a small, painless, raised skin papule, nodule, plaque, or edema that becomes indurated, crusted, and eventually ulcerates. The formation of the characteristic ulcer results from destruction of the subcutaneous adipose tissue leading to collapse of the epidermis. Over a period of weeks, the ulcer enlarges, with widespread thickening of subcutaneous tissue. Subsequently, there is necrosis that produces the characteristic undermining of the ulcer margins. In larger lesions, there may be extensive induration adjacent to the ulcer. Beyond this area of thickening, however, the skin appears normal, with no cellulitis or lymphadenopathy. Necrosis may extend down to muscle fascia and even to bone, but actual osteomyelitis is rare. Advanced lesions display massive tissue destruction and minimal inflammation, with extracellular microcolonies of *M. ulcerans* in the superficial necrotic areas [5, 6]. Characteristically, the lesions are not associated with pain or tenderness. The disease is rarely fatal but can lead to significant debilitation as well as enormous social and economic consequences in afflicted persons [2]. BU is most prevalent among the age group 5–15 years but is uncommon among neonates [1]. The exact mode of transmission and the incubation period of the causative organism of the disease are not known. We report cases of confirmed BU disease in two human immunodeficiency virus (HIV)-unexposed, vaginally delivered term neonates from the Ashanti region of Ghana and discuss the implications for understanding the incubation period of *M. ulcerans*.

## Case presentation

The clinical characteristics of the two patients are presented in Table 1.

**Table 1:** Clinical characteristics of two Ghanaian neonates with Buruli ulcer disease

Characteristic	Patient 1	Patient 2
Community/region	Ejisu, Ashanti	Atwima Nwabiagya, Ashanti
Mode and location of delivery	Vaginally, hospital	Vaginal, home
BCG vaccination	Yes	Yes
Lesion site	Chest and neck	Left gluteus, left neck
Type of lesion, WHO category	Edema/ulcer, category 2	Ulcer, category 3 (on account of multiple lesions)
Age (in days) when lesion was first seen	14 days	4 days
Age of neonate at PCR confirmation of BU	5 weeks	4 weeks
Use of topical herbs	Yes	Yes
Temperature	36.3 °C	37.9 °C
Ulcer healing by end of antibiotic treatment	Yes	Yes
Development of contracture	Yes, corrective surgery planned	Yes, corrective surgery planned
Organism causing secondary bacterial infection of ulcer	*Staphylococcus aureus*	*Escherichia coli*

*BCG* Bacille Calmette-Guérin, *BU* Buruli ulcer, *PCR* Polymerase chain reaction, *WHO* World Health Organization

### Patient 1

**Fig. 1:**
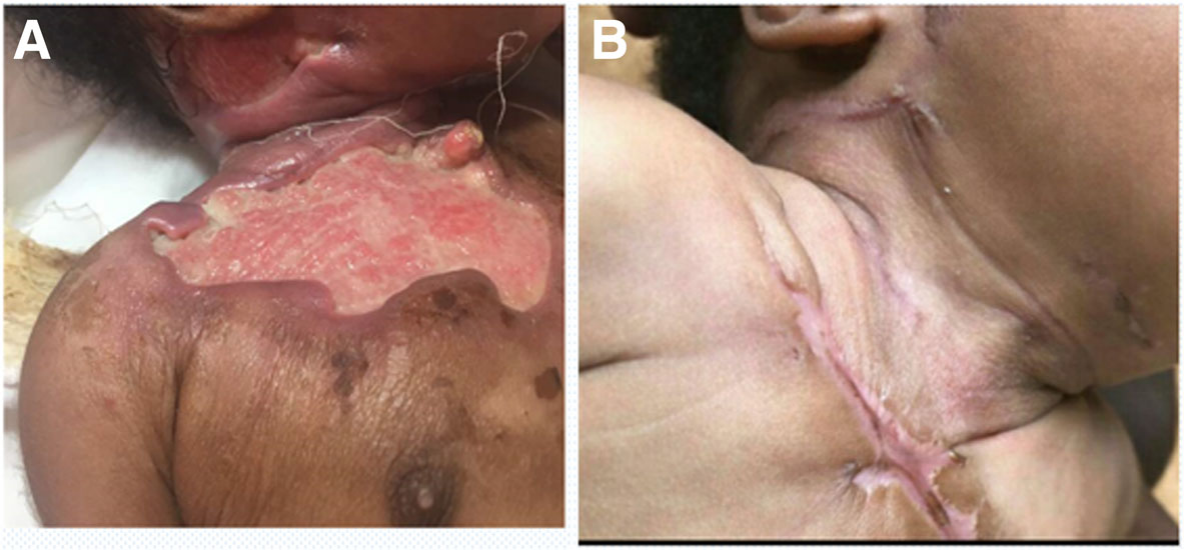
Pre- and post-treatment Buruli ulcer (BU) lesion in patient 1. Buruli ulcer located on the anterior chest of patient 1 before BU-specific antibiotic treatment (**a**). The ulcer had completely healed after 8 weeks of treatment with rifampicin and clarithromycin in addition to appropriate daily wound dressings (**b**)

This patient was from the Ashanti ethnic group in Ghana. Two weeks after hospital delivery, patient 1’s mother noticed a papule with associated edema on the right anterior chest wall and neck that later ulcerated and was associated with fever and poor feeding. Clinical examination revealed an 11-cm × 7-cm ulcer with undermined edges on the right anterior chest wall that extended to involve the right neck and pre- and postauricular regions and submental regions (Fig. 1). There was no restriction of neck movements. An initial clinical diagnosis of necrotizing fasciitis was made, and empiric treatment with gentamicin was started, based on the local culture and sensitivity patterns. The ulcer was secondarily infected by *Staphylococcus aureus*, which was sensitive to gentamicin. The diagnosis of BU was confirmed on the basis of a swab sample that was polymerase chain reaction (PCR)-positive for the IS2404 repeat sequence of *M. ulcerans*.

### Patient 2

**Fig. 2:**
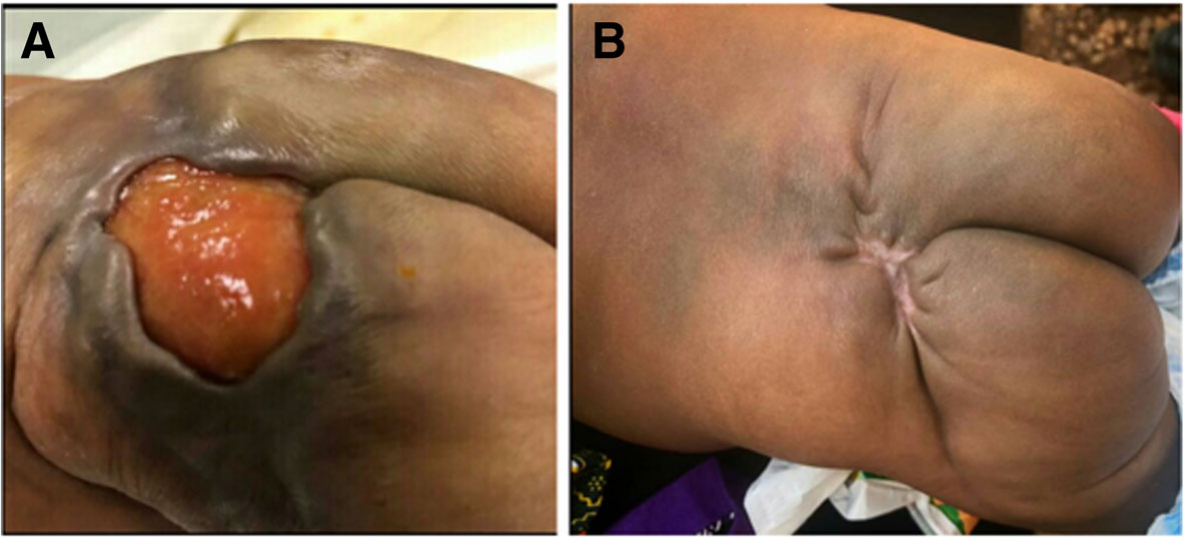
Pre- and post-treatment Buruli ulcer (BU) lesion in patient 2. Buruli ulcer on the gluteal region of patient 2 before BU-specific antibiotic treatment (**a**). The ulcer had completely healed after 8 weeks of treatment with rifampicin and clarithromycin in addition to appropriate daily wound dressings (**b**)

This patient was from the Ashanti ethnic group in Ghana. The mother of patient 2 reported noticing a swelling in the baby’s left gluteal region 4 days after birth. The lesion progressively increased in size to involve almost the entire left gluteal region. Around the same time, the mother noticed a second, smaller lesion on the forehead and left side of the neck. The patient had a 6 × 5-cm left gluteal ulcer with undermined edges, firm base, and floor covered with slough and purulent discharge (Fig. 2). An initial clinical diagnosis of necrotizing fasciitis was made, and empiric treatment with gentamicin was initiated. A wound swab culture result yielded *Escherichia coli*, which was sensitive to gentamicin. The diagnosis of BU was confirmed by PCR when the child was aged 4 weeks.

### Treatment

Both patients were treated with oral rifampicin and clarithromycin (at doses of 10 mg/kg and 15 mg/kg, respectively) administered daily for 8 weeks in addition to appropriate daily wound dressing. Antibiotic treatment was well tolerated by both patients. Treatment adherence was monitored using the standard Buruli ulcer 01 (BU 01) form used during the routine care of patients with BU. Their ulcers healed completely by the end of antibiotic treatment, but they developed contracted scars. Surgery to correct the deformities was planned by a plastic surgeon.

## Discussion

To the best of our knowledge, there has not been any previous report of confirmed BU in neonates in Ghana. Our report details two cases of PCR-confirmed BU in children whose lesions appeared at age 14 and 4 days, respectively. BU caused by *M. ulcerans* is endemic in parts of West Africa and usually presents as a painless subcutaneous nodule or plaque or as a more aggressive edematous lesion. Over time, all the lesions ulcerate and progressively enlarge. In Africa, the disease is most prevalent among the age group 5–15 years [1].

The mode of transmission of *M. ulcerans* infection is unknown, so the incubation period is difficult to estimate. Studies of people who have become infected after moving from a nonendemic to an endemic area have provided some insight into this. The Uganda Buruli Group studied refugees from Rwanda, where there are no reported cases, and found new Buruli lesions occurring 4–10 weeks after their arrival from an endemic area [7]. An incubation period of 6 weeks was estimated in a Nigerian physician who had visited an endemic area and later developed BU in New York [8]. In two recent studies among patients developing BU after a short visit to an endemic area in Australia, the median incubation periods were estimated as 135 and 143 days, respectively, with the shortest period given as 32 days [9, 10]. These studies provide a fair idea of the possible incubation period of BU in adults, but in neonates it may range from a few days to several weeks. A study of 13 cases of BU in Port Moresby identified the shortest known incubation period of 2–3 weeks in a 6-week-old baby born in an endemic region [11]. Cases of BU have been observed in 18-day-old [12] and 9-month-old [13] babies in the Democratic Republic of Congo and Uganda, respectively.

In the present study, lesions appeared 4 days after birth in patient 2. Unless infection was acquired *in utero*, this would be the shortest incubation period ever recorded. There was no evidence of BU in the mother, so it must be assumed that the baby was exposed to an *M. ulcerans-*contaminated water source shortly after birth. Because neither the mothers nor the babies in this report had HIV infection, the short incubation period probably resulted from immaturity of the neonatal immune system. Another explanation would be that the babies were exposed to a high infection dose, but there is no way of confirming this.

Bacille Calmette-Guérin (BCG) vaccine was thought to have a short-term protective effect against *M. ulcerans* infection in Uganda [14], and having a BCG vaccination scar was reported to provide significant protection against *M. ulcerans* osteomyelitis [15]. A recent study, however, did not find significant evidence of a protective effect of routine BCG vaccination on the risk of developing either BU or severe forms of BU [16]. The vaccine used now may not be exactly the same, and certainly it did not protect the babies in this report, who had both received it at birth.

Traditionally, combination antibiotic therapy with intramuscular streptomycin and oral rifampicin for 8 weeks has been used to treat BU disease, and this has been shown to give excellent cure rates [17, 18]. Due to the disadvantages of streptomycin [19] (prolonged intramuscular injections and risk of ototoxicity), daily oral treatment with rifampicin and clarithromycin is now recommended for treatment, especially in children.

There was evidence of symptomatic secondary bacterial infection in both the patients reported here, with *S. aureus* in patient 1 and *E. coli* in patient 2. This would account for the fever and poor feeding reported by both mothers and the systemic signs of infection that are not associated with BU. However, the ulcers did not start to heal until specific therapy for *M. ulcerans* was administered. Secondary bacterial infections with *S. aureus* and *E. coli* have been reported as common isolates, usually sensitive to gentamicin, as was the case in our patients [20].

## Conclusions

This report presents the first cases of BU in neonates from Ghana, a BU-endemic country in West Africa. Oral treatment with rifampicin and clarithromycin administered daily for 8 weeks proved effective in the management of BU in these neonates, thereby avoiding the use of painful daily streptomycin injections with their associated ototoxicity. BU should be included in the differential diagnosis of neonates who present with characteristic lesions. The incubation period of BU in this age group is probably shorter than is reported for adults.

## Data Availability

The datasets obtained and analyzed during the current study are available from the corresponding author on reasonable request.

## Abbreviations

BCG: Bacille Calmette-Guérin
BU: Buruli ulcer
BU 01: Buruli ulcer 01 form
HIV: Human immunodeficiency virus
IP: Incubation period
PCR: Polymerase chain reaction

## References

[1] Amofah GK, Sagoe-Moses C, Adjei-Acquah C, Frimpong EH. Epidemiology of Buruli ulcer in Amansie West district, Ghana. Trans R Soc Trop Med Hyg. 1993;87(6):644–5. 10.1016/0035-9203(93)90272-r 8296362

[2] Asiedu K, Etuaful S. Socioeconomic implications of Buruli ulcer in Ghana: a three-year review. Am J Trop Med Hyg. 1998;59(6):1015–22. 10.4269/ajtmh.1998.59.1015 9886216

[3] Stinear TP, Mve-Obiang A, Small PL, Frigui W, Pryor MJ, Brosch R, *et al.* Giant plasmid-encoded polyketide synthases produce the macrolide toxin of *Mycobacterium ulcerans*. Proc Natl Acad Sci U S A. 2004;101(5):1345–9. 10.1073/pnas.0305877101 PMC337055 14736915

[4] Demangel C, Stinear TP, Cole ST. Buruli ulcer: reductive evolution enhances pathogenicity of *Mycobacterium ulcerans*. Nat Rev Microbiol. 2009;7(1):50–60. 10.1038/nrmicro2077 19079352

[5] Hayman J. Clinical features of *Mycobacterium ulcerans* infection. Australas J Dermatol. 1985;26(2):67–73. 10.1111/j.1440-0960.1985.tb01819.x 4084161

[6] Guarner J, Bartlett J, Whitney EA, Raghunathan PL, Stienstra Y, Asamoa K, *et al.* Histopathologic features of *Mycobacterium ulcerans* infection. Emerg Infect Dis. 2003;9(6):651–6. 10.3201/eid0906.020485 PMC3000137 12780997

[7] Uganda Buruli Group. Epidemiology of *Mycobacterium ulcerans* infection (Buruli ulcer) at Kinyara, Uganda. Trans R Soc Trop Med Hyg. 1971;65(6):763–75. 10.1016/0035-9203(71)90090-3 5157438

[8] Lindo SD, Daniels F Jr. Buruli ulcer in New York City. JAMA. 1974;228(9):1138–9. 4406435

[9] Loftus MJ, Trubiano JA, Tay EL, Lavender CJ, Globan M, Fyfe JAM, *et al.* The incubation period of Buruli ulcer (*Mycobacterium ulcerans* infection) in Victoria, Australia remains similar despite changing geographic distribution of disease. PLoS Negl Trop Dis. 2018;12(3):e0006323. 10.1371/journal.pntd.0006323 PMC5875870 29554096

[10] Trubiano JA, Lavender CJ, Fyfe JA, Bittmann S, Johnson PD. The incubation period of Buruli ulcer (*Mycobacterium ulcerans* infection). PLoS Negl Trop Dis. 2013;7(10):e2463. 10.1371/journal.pntd.0002463 PMC3789762 24098820

[11] Reid IS. *Mycobacterium ulcerans* infection: a report of 13 cases at the Port Moresby General Hospital, Papua. Med J Aust. 1967;1(9):427–31. 10.5694/j.1326-5377.1967.tb21363.x 5336860

[12] Kiiza AM, Wood PB. Buruli ulcer in an 18-day-old baby. Trop Dr. 2012;42(1):48. 10.1258/td.2011.110230 22080481

[13] Tsukagoshi S, Dehn TC. Buruli ulcer in a nine-month-old boy. Ann R Coll Surg Engl. 2012;94(7):e215–6. 10.1308/003588412X13373405385692 PMC3954272 23031753

[14] Smith PG, Revill WD, Lukwago E, Rykushin YP. The protective effect of BCG against *Mycobacterium ulcerans* disease: a controlled trial in an endemic area of Uganda. Trans R Soc Trop Med Hyg. 1976;70(5–6):449–57. 10.1016/0035-9203(76)90128-0 841647

[15] Portaels F, Aguiar J, Debacker M, Steunou C, Zinsou C, Guedenon A, *et al.* Prophylactic effect of *Mycobacterium bovis* BCG vaccination against osteomyelitis in children with *Mycobacterium ulcerans* disease (Buruli ulcer). Clin Diagn Lab Immunol. 2002;9(6):1389–91. 10.1128/CDLI.9.6.1389-1391.2002 PMC130119 12414782

[16] Phillips RO, Phanzu DM, Beissner M, Badziklou K, Luzolo EK, Sarfo FS, *et al.* Effectiveness of routine BCG vaccination on Buruli ulcer disease: a case-control study in the Democratic Republic of Congo, Ghana and Togo. PLoS Negl Trop Dis. 2015;9(1):e3457. 10.1371/journal.pntd.0003457 PMC4287572 25569674

[17] Sarfo FS, Phillips R, Asiedu K, Ampadu E, Bobi N, Adentwe E, *et al.* Clinical efficacy of combination of rifampin and streptomycin for treatment of *Mycobacterium ulcerans* disease. Antimicrob Agents Chemother. 2010;54(9):3678–85. 10.1128/AAC.00299-10 PMC2935024 20566765

[18] Phillips RO, Sarfo FS, Abass MK, Abotsi J, Wilson T, Forson M, *et al.* Clinical and bacteriological efficacy of rifampin-streptomycin combination for two weeks followed by rifampin and clarithromycin for six weeks for treatment of *Mycobacterium ulcerans* disease. Antimicrob Agents Chemother. 2014;58(2):1161–6. 10.1128/AAC.02165-13 PMC3910847 24323473

[19] Klis S, Stienstra Y, Phillips RO, Abass KM, Tuah W, van der Werf TS. Long term streptomycin toxicity in the treatment of Buruli ulcer: follow-up of participants in the BURULICO drug trial. PLoS Negl Trop Dis. 2014;8(3):e2739. 10.1371/journal.pntd.0002739 PMC3953024 24625583

[20] Yeboah-Manu D, Kpeli GS, Ruf MT, Asan-Ampah K, Quenin-Fosu K, Owusu-Mireku E, *et al.* Secondary bacterial infections of Buruli ulcer lesions before and after chemotherapy with streptomycin and rifampicin. PLoS Negl Trop Dis. 2013;7(5):e2191. 10.1371/journal.pntd.0002191 PMC3642065 23658847

